# Risk of Mortality From Esophageal Cancer Among US Poultry Workers, 1950−2019

**DOI:** 10.1002/ajim.23742

**Published:** 2025-06-10

**Authors:** Leanna Delhey, Christina Joshua, Jaimi L. Allen, Robert Delongchamp, Benjamin C. Amick, Wendy Nembhard

**Affiliations:** ^1^ Department of Epidemiology, Fay W. Boozman College of Public Health University of Arkansas for Medical Sciences Little Rock Arkansas USA; ^2^ Department of Epidemiology, School of Public Health University of Michigan Ann Arbor Michigan USA

**Keywords:** esophageal cancer, food industry workers, lifestyle factors, occupational exposure, poultry, poultry slaughtering

## Abstract

**Background:**

While research suggests poultry industry workers have an increased risk of cancer mortality, little is known about their risk of esophageal cancer mortality. We investigated the association between working with poultry and esophageal cancer mortality while concurrently investigating other occupational and nonoccupational risk factors amongst poultry industry workers.

**Methods:**

We conducted a case‐cohort analysis from a cohort of unionized workers in the United States (*N* = 46,816) and conducted follow‐up for mortality from 1950 to 2019 with the National Death Index. Cases were those who died of esophageal cancer and a sub‐cohort was randomly selected (*N* = 2666) for further analysis. We interviewed participants and relatives about their work and personal life. We used multivariable Cox proportional hazards regression to estimate the hazard of esophageal cancer mortality due to working with poultry among the full cohort and weighted regression for the sub‐cohort and those interviewed. We conducted exploratory analyses to estimate hazard ratios (HR) and 95% confidence intervals (CI) for each interview question, adjusted for confounders, and computed a false discovery rate (FDR).

**Results:**

In the full and sub‐cohort, working in a poultry plant was associated with an increased hazard of esophageal cancer mortality (HR = 1.62, 95% CI = 1.05, 2.50; and HR = 1.65, 95% CI = 1.03, 2.65, respectively). Among survey respondents, working in a poultry plant appeared to decrease the risk of esophageal cancer mortality (HR = 0.67; 95% CI = 0.34, 1.35).

**Conclusions:**

Working in poultry plants may increase the risk of death from esophageal cancer, but further research is needed to validate these findings and explore potential mechanisms.

## 1 Introduction

Previous studies have reported that slaughtering chickens increased the risk of exposure to oncogenic viruses and cancer mortality [1, 2, 3, 4]. The US is the world's leading producer of poultry meat (nearly 17% of global production) and the second leading poultry meat exporter [5]. As of 2020, the poultry industry employed roughly 2 million workers across 40 states in the United States [5]. Research shows poultry industry workers tasked with slaughtering, processing, or packing poultry animals have an increased risk of exposure to harmful viruses and microorganisms [6, 7, 8, 9, 10, 11]. Poultry industry workers can slaughter and process up to an average of 250,000 chickens in a single day, making exposure to these potentially harmful viruses highly likely during a work day [3]. Epidemiologic studies investigated lung, brain, and pancreatic cancer deaths within a cohort of poultry industry workers who slaughter chickens and identified a link between mortality and tumor‐inducing viruses [6, 7, 8, 9, 10, 11]. In one large cohort study, a significant increase in mortality risk was observed for cancer sites that included the pancreas, brain, buccal cavity and pharynx, cervix trachea, bronchus, lung, lymphoid leukemia, monocytic leukemia, and the lymphatic system, in poultry industry workers tasked with slaughtering and processing poultry animals compared to the general US population [11]. These observations emphasize the importance of etiologic studies to confirm and elucidate the pathways of increased risk of cancer mortality among poultry workers.

Several potential mechanisms are proposed for an increased esophageal cancer mortality rate among poultry workers. First, certain avian retroviruses and herpes viruses infect and initiate carcinogenesis in poultry animals [4, 11, 12]. Human infection may result from contact with live poultry, their blood, secretions, feces, raw meat, and eggs during poultry work [3, 8, 13, 14, 15, 16, 17, 18, 19]. Oncogenic viruses to which workers are commonly exposed include retroviruses such as avian leukosis/sarcoma viruses (ALSV), reticuloendotheliosis virus (REV), and Marek's disease virus (MDV) [20]. Second, there is also increased risk for exposure to chemical carcinogens (e.g., polycyclic aromatic hydrocarbons [PAHs], benzene, phthalates, heterocyclic amines, and nitrosamines) through occupational tasks related to wrapping and labeling of meat, smoking poultry meat, aerosols emitted during cooking/frying of meat, and nitrosamines from curing meat [2, 10, 21, 22, 23, 24, 25, 26, 27, 28, 29, 30, 31, 32, 33, 34, 35, 36, 37, 38]. A third possible explanation for higher rates of esophageal cancer among poultry plant workers is related to the increased prevalence of human papillomavirus (HPV) infections. Certain HPV strains are linked with both squamous cell carcinoma and adenocarcinoma of the esophagus in humans [39, 40, 41]. Poultry, meat, and fish handlers experience a high occurrence of warts caused by HPV [39, 40, 41]. Studies show that workers who carry out tasks such as slaughtering, eviscerating, or handling raw or frozen chickens have a significantly higher prevalence of warts and HPV than other workers in the same plants [42, 43]. Thus, poultry workers may be highly susceptible to HPV infection because of an unknown factor in meat that enhances viral replication [43, 44]. Fourth, it is possible that poultry workers are more likely to adopt lifestyles or behaviors, such as tobacco use, alcohol consumption, and unhealthy diet, that increase their risk for esophageal cancer mortality [41, 45, 46, 47].

Therefore, we sought to determine if there is an increased risk of esophageal cancer mortality among poultry industry workers using an established US cohort. We further sought to explore whether specific occupational factors (e.g., direct involvement with the slaughtering process) and lifestyle factors (e.g., tobacco use, diet) among poultry industry workers were associated with increased risk of esophageal cancer mortality.

## 2 Materials and Methods

### 2.1 Study Population

**Figure 1:**
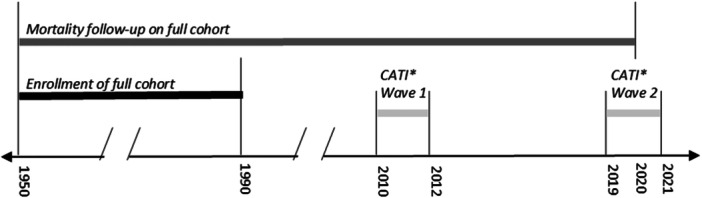
Study populations and data collection timeline. Black bar: All identified unionized members employed from January 1, 1950, to December 31, 1989 (full cohort). Dark gray bar: Follow‐up occurred from enrollment through death or December 31, 2019, to determine vital status. Light gray bar: *CATI‐Computer‐assisted telephone interviews. Wave 1 targeted all members in the sub‐cohort and cases identified as of 2010. Wave 2 targeted only cases. Sub‐cohort included those randomly selected from the full cohort. Cases included those who had died of esophageal cancer.

We conducted a case‐cohort study consisting of poultry and non‐poultry workers who were members of the following unions: (1) United Food and Commercial Workers (UFCW) meat cutter's union in Baltimore, Maryland, (2) Poultry Union in Marshall, Missouri, or (3) Union Pension Fund in Chicago, Illinois, and employed between January 1, 1950, and December 31, 1989 (Figure 1). These unions included workers from 23 poultry plants in seven states in the United States (Maryland [MD], Missouri [MO], Alaska, Arkansas, Louisiana, Maine, and Texas) and 39 non‐poultry plants in nine states (MD, Florida, Illinois [IL], Indiana, Massachusetts, New Jersey, Ohio, Pennsylvania, and Texas). Those working in the poultry plants likely completed tasks related to slaughtering, processing, or packing poultry animals, as well as sanitation and storage/shipping [6, 7, 8, 9, 10, 11, 48]. Non‐poultry industries included agriculture, cheese, meat (non‐poultry), seafood, drinks, general foods, Chinese foods, Hispanic foods, natural gas, office supplies, furniture, packaging, and a trucking company. Workers were initially identified from union records, as previously described [1, 2, 7, 10, 11]. From these records, study staff abstracted the worker's name, social security number, date of birth, sex, race/ethnicity (non‐Hispanic [NH] White or other race/ethnicity), date of hire, date of termination, plant name, and plant location. Using date of birth, we determined age in years at time of hire, termination, and death or censor date. Hereafter this population of union workers is referred to as the full cohort.

**Figure 2:**
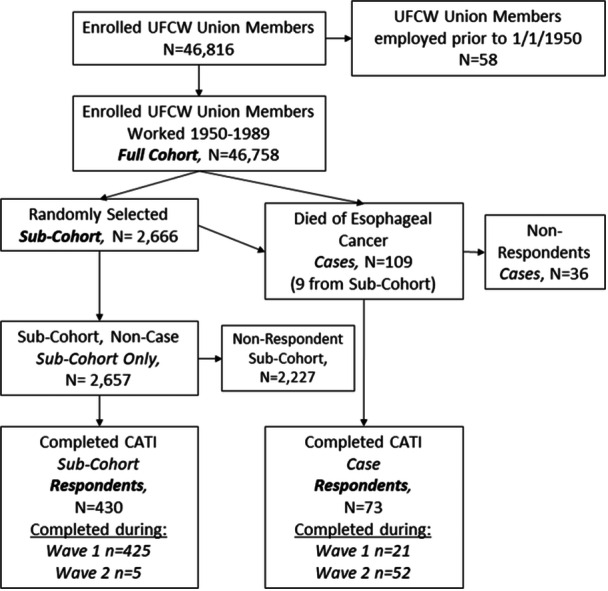
Study population flowchart. CATI, computer assisted interview; UFCW, United Food and Commercial Workers; Wave 1, 2010−2012, Wave 2, 2019−2021.

Study staff identified 46,816 working persons from the UFCW unions (poultry workers: 30,411; non‐poultry workers: 16,405). We excluded 58 workers who terminated employment before January 1, 1950, resulting in a full cohort of 46,758 workers (Figure 2). The randomly selected sub‐cohort included 2666 workers [2, 7, 10]. As of December 31, 2019, 109 persons had died of esophageal cancer and were included as cases; nine of these were previously selected into the sub‐cohort (Figure 2). The response rate in the sub‐cohort (non‐cases) was low, 430 out of 2657 (16%, Figure 2). Among cases, the response rate was moderate 73 out of 109 (67%, Figure 2).

### 2.2 Mortality Follow‐Up

Data from the National Death Index (NDI) were linked with existing data for the full cohort by various combinations of social security number, name, date of birth, sex, and last known state of residence from the start of the study through December 31, 2019 (Figure 1). From the NDI, we determined vital status, and where applicable, date of death, and primary cause of death (International Classification of Diseases, Ninth or Tenth Revision, Clinical Modification [ICD‐9‐CM or ICD‐10‐CM] codes). Cases consisted of all persons who died of esophageal cancer (ICD‐9‐CM code 150.* or ICD‐10‐CM code C15.*). We censored study subjects at date of death, 100 years of age, or the end of follow‐up period (December 31, 2019), whichever occurred first. We censored a small number of persons at 100 years of age (*n* = 17, 0.04% of the full cohort), because by this age, it is likely they have died, but we did not successfully match them with data from the NDI due to incomplete or inaccurate identifying information from union records.

### 2.3 Interview Follow‐Up

We collected additional information using a computer‐assisted telephone interview (CATI) about cases and those randomly selected from the base population of the full cohort as part of the initial case‐cohort study conducted in this study population (henceforth known as the sub‐cohort, Figures 1 and 2) [2]. For those who were deceased at the time of the interview (all cases and some of the sub‐cohort), interviewers contacted the next of kin to complete the CATI. A small validation study was previously conducted to determine the reliability between CATI responses by next‐of‐kin compared to self‐reports by living subjects. The investigators found high agreement between responses: ≥ 90% agreement for 62% of questions, ≥ 80% agreement for 81% of questions, and ≥ 70% agreement for 96% of questions [10].

Attempts to trace/contact participants occurred in two waves (2010−2012 and 2019−2021, Figure 1). In wave one, research staff traced/contacted members or the next of kin of the sub‐cohort and cases identified via NDI as of 2010. In wave two, research staff traced/contacted next‐of‐kin for both newly identified cases and cases not successfully interviewed in wave one. Hereafter, those successfully interviewed are referred to as respondents (Figure 2). Tracing of study participants was performed using a variety of sources: Interactive Data LLC (idiCORE), Thomson Reuters (CLEAR), Microbilt, Lexis/Nexis, Skip Tracing Inc., Whitepages Inc., Skip Genie (The Tracer), Intelius.com, findagrave.com, Ancestry.com, the United States Census Bureau (www.census.gov), as well as searching obituaries, social media profiles, and company profiles identified through online search engines (e.g., www.Google.com). After successful contact, research staff confirmed identity/relation to the subject, obtained verbal consent, and conducted the CATI to obtain detailed information on potential occupational and nonoccupational exposures. Details on the CATI questions are published elsewhere [1, 10]. Briefly, the CATI included questions related to occupational factors like the work environment and specific job details in addition to lifestyle factors like tobacco, alcohol, and substance use, diet, medical history, home environment, physical activity, hobbies, and general lifestyle.

### 2.4 Statistical Analysis

We conducted a literature review to identify risk factors for esophageal cancer and esophageal cancer mortality and to consider potential associations with working in a poultry plant. We used www.daggity.net to create a directed acyclic graph (DAG) and identify the potential confounders that would need to be controlled for to evaluate the association between poultry work exposures and risk of esophageal cancer mortality [49, 50]. Daggity.net uses causal diagram analysis and algorithms previously described by Textor and Liśkiewicz [50] to identify minimal covariate adjustment for potential bias due to confounding. We then selected data from union records or the CATI that would be representative of these indicated confounders.

We calculated descriptive statistics to describe the demographics, union characteristics, cause of death, the time period attempted contact for the full cohort, the sub‐cohort, cases, and the respondents. Among the respondents, descriptive statistics were computed to describe the additional identified confounders from the CATI. Categorical variables were described using frequency and percentages. Continuous variables were described using means and standard deviations. t‐test and chi‐square tests were conducted to determine whether continuous and categorical characteristics, respectively, differed. When expected counts for a cell were ≤ 5 for ≥ 20% of the cells, we collapsed categories and if needed used Fisher's exact test. We evaluated differences among the sub‐cohort and esophageal cases for the respondents versus those not interviewed. Differences were also evaluated among the respondents, between those who died of esophageal cancer and those in the sub‐cohort who did not die of esophageal cancer.

We fit Cox proportional‐hazard regression models to estimate the hazard ratio (HR) and 95% confidence intervals (CI) foresophageal cancer mortality for working at a poultry plant relative to working at non‐poultry industrial plants. Age was used as the time to event variable, beginning with age at hire and ending with follow‐up at age of death from esophageal cancer or age at time of censoring (death from other causes, 100 years old, or end of study). First, we fit a model adjusted for confounders available within the union records (sex, race, union, and decade of hire). This was done for the full cohort, sub‐cohort, and respondents. Cox regression models were then computed only for respondents and adjusted for all confounders as identified by the DAG with available data from the CATI.

In the analysis of the sub‐cohort and respondents, we applied weights to account for the case‐cohort design and to estimate HR and CI reflective of the full cohort. The probability of selection for members of this sub‐cohort is π_i _= selected/full cohort = 2666/46,758 = 0.057. All cases were selected; hence, their probability of selection is π_i _= 1. Ideally, all cases and all sub‐cohort members are queried and their responses can be analyzed by a weighted Cox regression where the weights are the inverse of the probability of selection, *w*_*i*_ = 1/*π*_*i*_ [51, 52]. There are other weighting schemes in the literature, but we chose to analyze these data as a survey sample of the full cohort, since these weights can be further adjusted for nonresponse [52, 53, 54]. If *ϕ*_*i*_ is the probability that a subject responds to our query, then the adjusted weight is *w*_*i*_ = 1/(*ϕ*_*i*_ *π*_*i*_). We adjusted for nonresponse bias by using post‐stratification methods. We computed *ϕ*_*i*_ as the proportion of successful interviews within strata based on sex, race (White or non‐White), decade of hire (1950, 1960, or 1970), and union (MO, MD, and IL).

We conducted analyses to identify the HR and CI of esophageal cancer mortality for each question from the CATI, again using age for the time‐to‐event variable. Questions were grouped by the nature of the answers (yes/no, how often, how long, and in what decade the activity/item began). We then filtered the questions to remove questions for which fewer than 100 participants responded or questions where a category contained over 90% of the responses due to the likelihood of being unable to obtain a valid HR from these responses. All analyses used the weights for the interviewed as described above and were first adjusted for race, sex, union, and decade of hire, and then for all confounders identified from the DAG where available. To determine which associations were most likely to be true, we computed a false discovery rate (FDR) [55]. Because many of the questions in the survey are related, the FDR is believed to overestimate the proportion of false positive results.

### 2.5 Ethics Review and Approval

The original cohort study was reviewed and approved by the Institutional Review Board, University of North Texas Health Science Center, and the follow‐up case‐cohort study was reviewed and approved by the Institutional Review Board, University of Arkansas for Medical Sciences. Before participating, all respondents provided verbal consent agreeing to the CATI to provide information about the participant or the deceased person as appropriate.

## 3 Results

**Figure 3:**
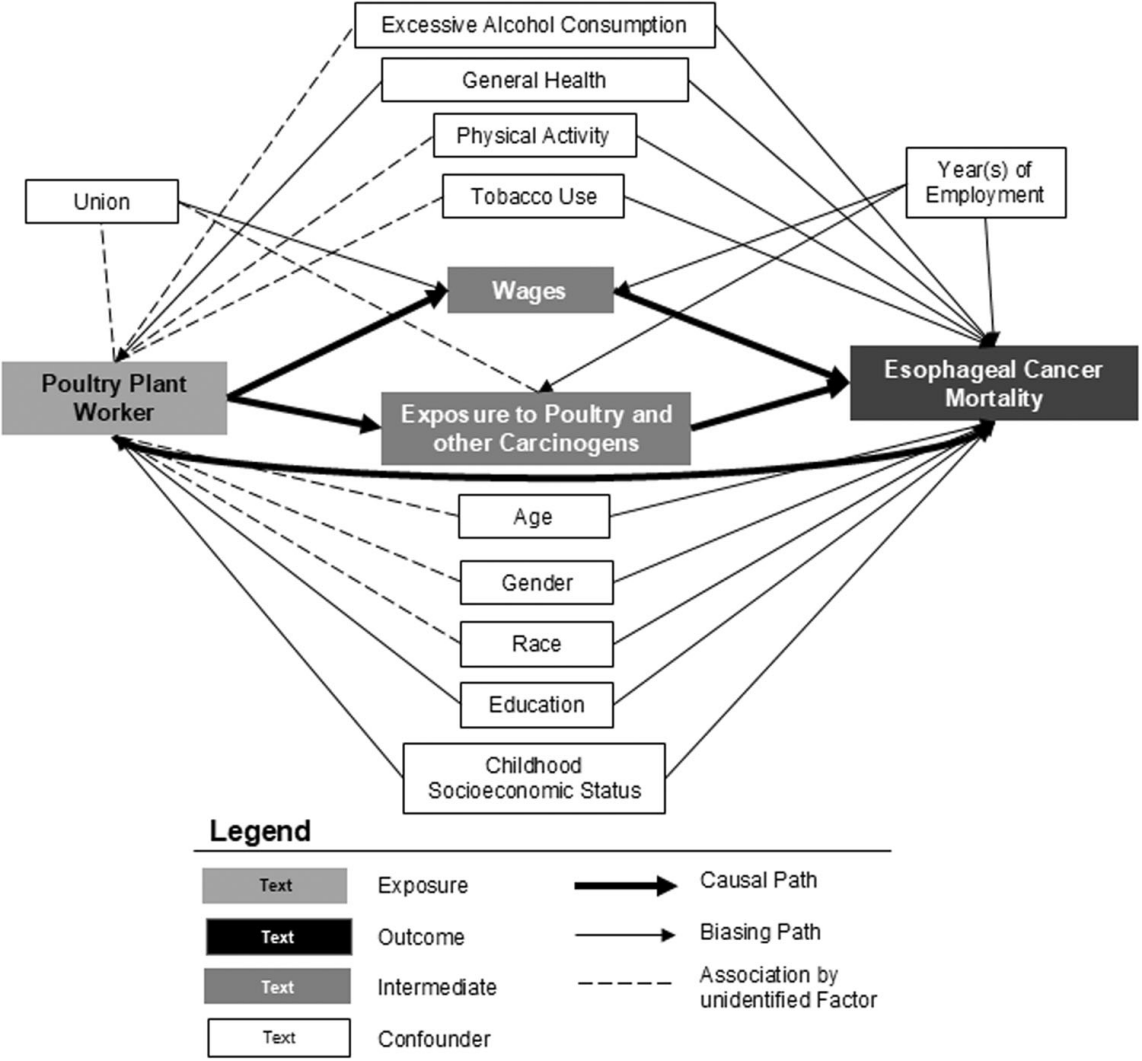
Directed acyclic graph (DAG) of the effect of poultry exposure on esophageal cancer mortality.

Figure 3 portrays the DAG created from our review of the literature. Identified risk factors for esophageal cancer mortality included age, sex, race, socioeconomic status (during childhood and adulthood), general health or comorbid conditions, and health behaviors such as tobacco use, alcohol use, diet, and physical activity [45, 46, 47, 56, 57]. These risk factors may be related to the mechanism for developing cancer (e.g., health behaviors) or related to the risk of mortality due to cancer (e.g., race). Some of these risk factors are likely to directly influence working in the poultry industry (e.g., education and health will influence your choice of work), while others may be associated due to an unidentified factor such as the work culture at the plant and its appeal to certain workers (e.g., current plant workers are accepting of tobacco use). We also identified union and decade of employment as likely be associated with being a poultry plant worker, with intermediate variables (exposure to poultry carcinogens and wages), and esophageal cancer mortality due to differences in regulations by union/state and time. The minimum identified confounders included: age (calculated from birth date and date of hire), sex (union records: male or female), race/ethnicity (union records: non‐Hispanic white or other), education (no measure available), childhood socioeconomic status (no measure available), tobacco use (ever used any type of tobacco), excessive alcohol use (reported ever being drunk on at least five occasions), physical activity (reported regular exercise for most of adult life), medication use as a proxy for general health (reported taking any medications on weekly basis for more than a year), union (Pension Fund of Chicago; Poultry Union, MO; Meat cutter's Union, MD), and year(s) of employment (decade of hire date: 1950s−1980s).

Table 1 provides a summary of union and demographic characteristics for the full cohort (*N* = 46,758), sub‐cohort (*N* = 2666), and esophageal cancer fatalities (*N* = 109). At the time of hire, workers had a median age of 25 years with 93% being 50 years old or younger, 48% male, and 74% NH White. Most were members of the Union Pension Fund of Chicago (65%) and worked in the poultry industry (65%) and the remaining worked in industries related to seafood (14%), meat (4%), general foods (7%), or other industries (10%). As of December 31, 2019, 15,311 persons died of various causes (esophageal cancer: 109 (0.23%); other type of cancer: 2,909 (6.22%); non‐cancer cause: 15,202 (32.51%) with an average of 37.88 years of follow‐up time). Among the sub‐cohort, those interviewed relative to those not interviewed were younger and more likely to be poultry workers, belong to the Union Pension Fund of Chicago, had died of cancer, had slightly longer follow‐up, and were contacted/traced within the 2010–2012 period. Among the cases, those interviewed relative to those not interviewed were more likely to have belonged to the Pension Fund of Chicago and less likely to be a part of the UFCW meat cutter's union in Baltimore, MD, or have been contacted/traced in both time periods.

**Table 1:** Union and demographic characteristics of the full‐cohort, sub‐cohort, and esophageal cancer fatalities.

Characteristic	Full‐cohort	Sub‐cohort			Esophageal cancer fatalities		
	(*N* = 46,758)	Overall (*N* = 2666)	Respondents (*N* = 437)	*p* value	Overall (*N* = 109)	Respondents (*N* = 73)	*p* value
Age (years)^a^	29.21 (11.28)	29.13 (11.08)	27.92 (9.69)	0.0058	30.68 (11.83)	30.88 (11.04)	0.8016
Age group^b^							
≤ 50 years old	43,355 (92.72%)	2493 (93.51%)	421 (96.34%)		100 (91.74%)	69 (94.52%)	
> 50 years old	3403 (7.28%)	173 (6.49%)	16 (3.66%)	0.0087	9 (8.26%)	4 (5.48%)	0.1531^c^
Gender^b^							
Female	24,538 (52.48%)	1384 (51.91%)	238 (54.46%)		20 (18.35%)	15 (20.55%)	
Male	22,220 (47.52%)	1282 (48.09%)	199 (45.54%)	0.2434	89 (81.65%)	58 (79.45%)	0.3982
Race^b^							
White	34,459 (73.70%)	1962 (73.59%)	316 (72.31%)		68 (62.39%)	48 (65.75%)	
Non‐White	12,299 (26.30%)	704 (26.41%)	121 (27.69%)	0.6030	41 (37.61%)	25 (34.25%)	0.0709
Plant industry^b^							
Poultry	30,411 (65.04%)	1753 (65.75%)	337 (77.12%)		64 (58.72%)	39 (53.42%)	
Non‐poultry	16,347 (34.96%)	913 (34.25%)	100 (22.88%)	< 0.0001	45 (41.28%)	34 (46.58%)	0.1101
Non‐poultry industries							
Seafood	6562 (14.03%)	375 (14.07%)	40 (9.15%)		22 (20.18%)	15 (20.55%)	
Meat	1840 (3.94%)	101 (3.79%)	1 (0.23%)		7 (6.42%)	5 (6.85%)	
General foods	3463 (7.41%)	205 (7.69%)	32 (7.32%)		7 (6.42%)	7 (9.59%)	
Other	4482 (9.59%)	232 (8.70%)	27 (6.18%)		9 (28.44%)	7 (9.59%)	
UFCW union^b^							
Pension Fund of Chicago	30,488 (65.20%)	1776 (66.62%)	332 (75.97%)		56 (51.38%)	47 (64.38%)	
Poultry Union, MO	7700 (16.47%)	432 (16.20%)	69 (15.79%)		9 (8.26%)	6 (8.22%)	
Meat Cutter's Union, MD	8570 (18.33%)	458 (17.18%)	36 (8.24%)	< 0.0001	44 (40.37%)	20 (27.40%)	0.0003
Cause of death^b^							
Esophageal cancer	109 (0.23%)	9 (0.34%)	7 (1.60%)		109 (100%)	73 (100%)	
Other cancer site	2909 (6.22%)	171 (6.41%)	54 (12.36%)		0 (0%)	0 (0%)	
Non‐cancer cause	15,202 (32.51%)	826 (30.98%)	74 (16.93%)		0 (0%)	0 (0%)	
Presumed alive	26,878 (60.96%)	1660 (62.27%)	302 (69.11%)	< 0.0001	0 (0%)	0 (0%)	n/a
Contact/trace attempt time^b^							
2010−2012 only	n/a	2651 (99.44%)	428 (97.94%)		21 (19.27%)	21 (28.77%)	
2019−2021 only		0 (0%)	0 (0%)		31 (28.44%)	25 (34.25%)	
2010−2012 and 2019−2021		15 (0.56%)	9 (2.06%)	0.0002^c^	57 (52.29%)	27 (36.99%)	< 0.0001

^a^ Mean (standard deviation), *p*‐value for *t*‐test.

^b^ *N* (%), *p*‐value for chi‐square test unless otherwise indicated.

^c^ *p*‐value for Fisher's exact test as > 20% of cells have expected counts < 5.

Table 2 displays a comparison of key characteristics (exposure and likely confounders) as identified using the DAG (Figure 3) and available within the data for the respondents by whether they died of esophageal cancer or not. Esophageal cancer cases were on average older and more likely to be male and belonged to the UFCW meat cutter's union in Baltimore, MD. Esophageal cancer cases were less likely to have worked in the poultry industry (53.42% vs. 77.44%). Esophageal cancer cases had a higher prevalence of persons who reported ever using tobacco, ever using alcohol, and ever being drunk on five or more occasions. Esophageal cancer cases had a lower prevalence of persons who reported weekly consumption of fruits and vegetables and exercised regularly.

**Table 2:** Comparison of identified confounders between those who died of esophageal cancer and those who did not among respondents.

Characteristic	Sub‐cohort^a^ (*N* = 430)	esophageal cancer^b^ (*N* = 73)	*p* value
Age (years)^c^	27.93 (9.69)	30.88 (11.04)	0.0191
Age group^d^			
≤ 50 years old	414 (96.28%)	69 (94.52%)	
> 50 years old	16 (3.72%)	4 (5.48%)	0.5127
Gender^e^			
Female	237 (55.12%)	15 (20.55%)	
Male	193 (44.88%)	58 (79.45%)	< 0.0001
Race^e^			
White	310 (72.43%)	42 (73.68%)	
Non‐White	118 (27.57%)	15 (26.32%)	0.8419
Plant industry^e^			
Poultry	333 (77.44%)	39 (53.42%)	
Non‐poultry	97 (22.56%)	34 (46.58%)	< 0.0001
UFCW Union^e^			
Chicago Pension Fund	325 (75.58%)	47 (64.38%)	
Missouri Union	69 (16.05%)	6 (8.22%)	
Maryland Union	36 (8.37%)	20 (27.40%)	< 0.0001
Tobacco use^e^, ^f^			
Ever	284 (66.05%)	55 (75.34%)	
Never	143 (33.26%)	15 (20.55%)	
Unknown	3 (0.70%)	3 (4.11%)	0.0446
Alcohol intoxication^e^, ^i^			
Ever	155 (36.05%)	31 (42.47%)	
Never	257 (59.77%)	29 (39.735)	
Unknown	18 (4.19%)	13 (17.81%)	0.0375
Regular exercise^e^			
Yes	281 (65.35%)	33 (45.21%)	
No	143 (33.26%)	34 (46.58%)	
Unknown	6 (1.40%)	6 (8.22%)	0.0070
Weekly medication use^e^, ^k^			
Yes	242 (56.28%)	23 (31.51%)	
No	167 (38.84%)	27 (36.99%)	
Unknown	21 (4.88%)	23 (31.51%)	0.0752

^a^ Respondents within the sub‐cohort excluding seven who died of esophageal cancer.

^b^ Respondents within cases of esophageal cancer mortality.

^c^ Mean (standard deviation), *p*‐value for *t*‐test.

^d^ N (%), *p*‐value for Fisher's exact test as > 20% of cells have expected counts < 5.

^e^ N (%), *p*‐value for chi‐square test.

^f^ Ever used any form of tobacco (includes cigarette, chewing, pipe, or cigar).

^g^ For chi‐square test, responses were combined resulting in following categories: None, < 10/week, 10−19/week, ≥ 20/week.

^h^ Ever used any form of alcohol (includes beer, wine, or liquor)

^i^ Ever been drunk on more than five occasions.

^j^ For chi‐square test, frequencies were combined resulting in following categories: < 1/week (includes never), every week

^k^ Ever take at least one medication each week indicating chronic illness.

**Figure 4:**
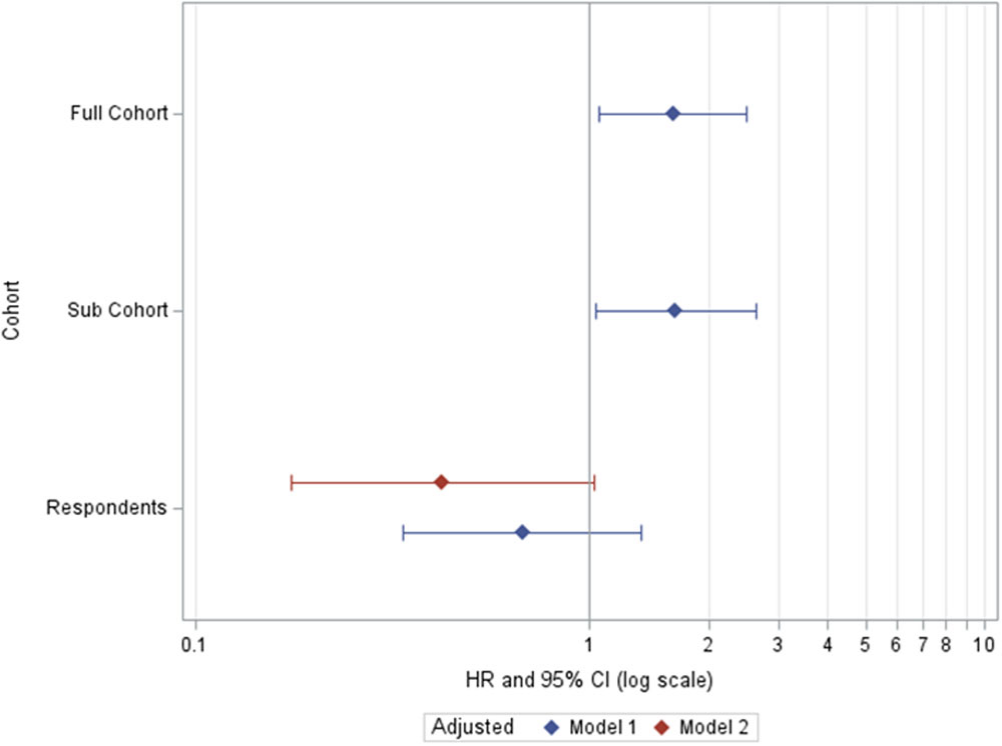
Hazard ratios and 95% confidence intervals for esophageal cancer mortality associated with working in the poultry industry. Model 1 is adjusted for age, gender, and race. Model 2 is adjusted for age, sex, race, tobacco use, alcohol intoxication, regular exercise, and weekly medication use.

Table 3 and Figure 4 display the estimated HRs and CIs around these ratios for the full cohort, sub‐cohort, and respondents. In the full cohort, a 62% increased rate of esophageal cancer mortality was observed among poultry compared to nonpoultry workers (HR = 1.62; 95% CI = 1.05, 2.50). The case‐cohort analysis of the sub‐cohort essentially replicates the results for the full cohort (HR = 1.65; 95% CI = 1.03, 2.65). Despite post‐stratification weighting to account for potential response bias, the case‐cohort analyses of the respondents did not demonstrate an increased hazard of esophageal cancer associated with working with poultry (HR = 0.67; 95% CI = 0.34, 1.35). We obtained similar results regardless of the strata used for post‐stratification weighting. For the respondents, the complete model adjusting for identified confounders minimally altered these results and the HR remained less than 1.00 (HR = 0.42; 95% CI = 0.18, 1.02).

**Table 3:** Cox regression models fit to determine the hazard ratio of esophageal cancer mortality for working at a poultry plant relative to working at plants within other industries.

Parameter	Full cohort *N* = 46,758 (event = 109)	Sub‐cohort *N* = 2766 (event = 109)	Respondents *N* = 503 (event = 73)	Respondents–fully adjusted *N* = 442 (event = 46)
Poultry worker				
Yes	1.62 (1.05, 2.50)	1.65 (1.03, 2.65)	0.67 (0.34, 1.35)	0.42 (0.18, 1.02)
No	Ref	Ref	Ref	Ref
Sex				
Male	6.05 (3.68, 9.95)	5.66 (3.39, 9.45)	5.31 (2.61, 10.80)	6.20 (2.24, 17.15)
Female	Ref	Ref	Ref	Ref
Race				
Other				1.26 (0.46, 3.50)
Race/ethnicity				Ref
Non‐Hispanic	1.16 (0.76, 1.79)	1.17 (0.73, 1.89)	1.10 (0.51, 2.38)	
White	Ref	Ref	Ref	
Union				
Missouri Union	0.71 (0.34, 1.51)	0.79 (0.35, 1.76)	0.94 (0.29, 3.05)	1.34 (0.34, 5.33)
Maryland Union	2.21 (1.22, 4.00)	2.65 (1.24, 5.65)	1.22 (0.33, 4.48)	0.66 (0.12, 3.59)
Chicago pension fund	Ref	Ref	Ref	Ref
Decade of Hire				
1950s	1.11 (0.43, 2.86)	0.91 (0.32, 2.59)	3.85 (0.51, 29.22)	8.11 (0.51, 128.13)
1960s	1.15 (0.49, 2.72)	1.20 (0.43, 3.32)	1.29 (0.27, 6.10)	2.74 (0.38, 19.78)
1970s	1.58 (0.84, 2.99)	1.56 (0.81, 3.00)	1.38 (0.59, 3.24)	1.88 (0.62, 5.69)
1980s	Ref	Ref	Ref	Ref
Tobacco user^a^				
Ever				1.14 (0.44, 2.97)
Never	N/A	N/A	N/A	Ref
Alcohol intoxication^b^				
Yes				1.41 (0.65, 3.04)
No	N/A	N/A	N/A	Ref
Regular exercise				
Yes				0.95 (0.44, 2.09)
No	N/A	N/A	N/A	Ref
Weekly medication use^c^				
Yes				0.32 (0.15, 0.71)
No	N/A	N/A	N/A	Ref

^a^ Ever used any form of tobacco (includes cigarette, chewing, pipe, or cigar).

^b^ Ever been drunk on more than five occasions.

^c^ Ever take at least one medication each week indicating chronic illness.

**Figure 5:**
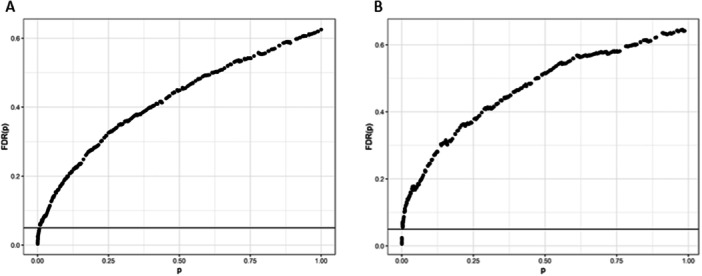
*p* value plot for the obtained hazard associated with each interview question and esophageal cancer mortality. (A) Adjusted for age, sex, and race. (B) Adjusted for age, sex, race, tobacco use, alcohol intoxication, regular exercise, and weekly medication use.

After applying filter criteria (e.g., at least 100 respondents, no response level with > 90% of respondents), we retained 446 questions. We determined the HR for esophageal cancer mortality after adjusting for age, sex, and race (Table SI). Figure 5A shows the *p*‐value plot for these questions with $\hat{\omega }=0.625$, for the proportion of true null hypotheses. Thirty‐five questions are in the set with an FDR < 0.05; all but two are true effects. We note that yes to “ever been treated with radiation” and “cancer” are among the predictors of death from esophageal cancer (Table SI; HR = 14.07; 95% CI = 5.89, 33.58 and HR = 8.08; 95% CI = 3.42, 19.09; respectively). Poultry‐associated jobs (e.g., working with chickens, working at chicken processing plant, and direct contact with chicken blood) are also among the 35; however, they have HRs < 1.00, contrary to our hypothesis (Table SI). We repeated the above analyses to additionally adjust for tobacco (ever used vs. never used), excessive alcohol use (reported they were drunk on at least five occasions or not), regular exercise as adult (yes/no), and weekly medication use (a possible indicator of chronic disease, yes weekly use of medication or no). Only six of 415 questions had an FDR < 0.05, two of which were radiation treatment and cancer (Figure 5B; Table SI; HR = 17.86; 95% CI = 5.15, 61.89 and HR = 15.19; 95% CI = 4.74, 48.76; respectively).

## 4 Discussion

Among the full cohort, working in the poultry industry is associated with a 60% increased risk of esophageal cancer mortality compared to working in other similar food or commercial industries. Notably, previous research in this area had not investigated esophageal cancer mortality, focusing instead on other cancer types or health outcomes [1, 2, 3, 7, 8, 10, 11]. Among the respondents, we did not observe this increased risk of esophageal cancer mortality for poultry workers or with specific job tasks likely to expose workers to oncogenic viruses (e.g., slaughtering). Additionally, lifestyle factors, such as excessive drinking and smoking, did not have an observed effect on the risk of esophageal cancer mortality. This may be due to responder bias and differential misclassification in the interview questions as discussed below.

This study has several strengths. It used a case‐cohort design with a known population (UFCW unionized workers). The exposed and unexposed workers all came from this UFCW population and were likely similar in many confounders such as age, gender, socioeconomic status, health, and behaviors. This reduced potential confounding in the analyses of the full cohort and sub‐cohort. We were also able to consider potential selection bias by comparing the obtained HR among the interviewed with the full cohort and sub‐cohort. This allowed us to assess the validity of our results among the interviewed and the potential results observed in the analyses of specific job tasks and lifestyle characteristics. This study is generalizable to the US since the unions included members from multiple states across the US; however, the cohort was recruited from 1950 to 1989 and may not be generalizable to current years. In recent decades, changes may have been made to better protect poultry workers from harmful environments, and a larger proportion of food industry laborers identify as Hispanic/Latino [58, 59]. Additionally, these findings may not be generalizable to non‐unionized workers who may experience different working environments.

The analysis of the respondents displayed in Table 3 and Figure 4 used weights derived from post‐stratification with available covariates. These weights assume that the interviewed sub‐population is a random sample of the full cohort within each stratum. In addition to the weights described in this paper, we also considered other weights which considered decade of death, wave of attempting CATI, or how long it was after death that we attempted the CATI (grouped by 5‐year periods and those alive were set to 0). None of these weights appreciably improved the concordance between HRs among the respondents and the full cohort. Ideally, everyone in the sub‐cohort and the cases would have been interviewed; however, due to difficulty in tracing, locating, and contacting subjects and next of kin, the participation rate was low (18% among the sub‐cohort, 67% among cases). Selection bias in the respondent cohort is very possible. Respondents differed from nonrespondents on measured characteristics (Table 2) and possibly unmeasured characteristics (e.g., socioeconomic status). The differences were most likely differential based on the exposure: if they worked in the poultry industry, and the outcome: mortality, and the cause of death. This is supported by the positive HRs observed in the full and sub‐cohorts, which would not be biased by nonresponse (Figure 4). We attempted to account for selection bias using weights within strata defined by known characteristics; however, this assumes respondents within each strata reflect the full cohort. This assumption does not appear to be true as obtained HRs were very different for the respondents (Figure 4). Selection bias may persist related to characteristics we were unable to account for (e.g., socioeconomic status).

Furthermore, analyses of interview questions within the respondent cohort may have been biased by differential exposure misclassification. Ideally, we would have obtained this information prospectively from union records (e.g., job title or descriptions) or directly from the enrolled union members. As this was not feasible, we instead had to conduct interviews retrospectively after cases were deceased. Since cases died from esophageal cancer, information about their exposure was obtained from their next of kin or a friend (proxy respondent). In contrast, controls could be deceased or living at the time of the interview; if living, the control was interviewed about their exposure. The accuracy of interview responses likely depended on whether the control was alive, the length of time since the death of the case or control, and how well the proxy respondent knew the subject's occupational exposure history or job duties. In the worst‐case scenario, proxy‐respondents may not have known or recalled information about the subject's exposure history very well. In contrast, respondents within the sub‐cohort who self‐report would more accurately recall their occupational exposure history and job duties; however, the previously mentioned validation study suggests high agreement between self‐report and next‐of‐kin responses [10]. It is also possible that respondents for those who worked in the poultry industry or who died of esophageal cancer may be more likely to report accurately as this study may be more relevant to them. Since this differential misclassification is likely minor, we expect it would bias the risk estimate toward the null.

Additionally, confounding may have influenced the effect of working in the poultry industry and exposure to interview questions in our analyses. In the full cohort and sub‐cohort, we were only able to control for information obtained from union records and our findings may have been confounded by health and other health behaviors like smoking, alcohol use, and physical activity (Figure 1) [45, 46, 47]. Additionally, in all cohorts, we were unable to control for factors like childhood socioeconomic status and education as this information was not obtained in the CATI or union records. It is well established that childhood socioeconomic status influences adult educational attainment and occupation [56, 60]. Adult‐level education is also well known to influence occupation [61, 62]. Both childhood socioeconomic status and education are associated with poor health outcomes, and likely to influence esophageal cancer mortality [63, 64, 65]. We also note that some bias due to confounding is partly controlled by study design, as the unexposed population consisted of workers from similar plants of different industries (meat, seafood, general food plants) who likely had similar characteristics related to socioeconomic status, education, and health. The direction of the residual confounding is unknown as it is not clear how factors like tobacco use, exercise, and general health may differ between those who work in the poultry industry and those who work in other types of industries.

We were also unable to investigate other outcomes including esophageal cancer incidence, or consider cancer subtypes due to a lack of access to this information. It is important to note that the conclusions regarding esophageal cancer mortality may not necessarily apply to esophageal cancer incidence. In the past decades, survival rates for esophageal cancer have improved, likely due to advances in screening and treatments [66]. Workers who developed esophageal cancer but survived would not be identified as cases. By focusing on mortality, this study may only identify the most severe cases or those least likely to be screened or receive adequate treatment. Furthermore, one of our hypothesized mechanisms for the increased risk of esophageal cancer was HPV‐related. It would have been interesting to explore how many of those with esophageal cancer were diagnosed with squamous cell carcinoma versus adenocarcinoma [39, 40, 41]. By leveraging advancements in the national program of cancer registries since the 1990s, future research could more comprehensively assess esophageal cancer incidence and subtypes among poultry workers to allow for a better understanding of their potential increased risk and the underlying mechanisms [67].

In conclusion, this study provides novel evidence that working in the poultry industry may be associated with an increased risk of death from esophageal cancer as demonstrated in the full cohort. While residual confounding remains a possibility, and the precise mechanisms driving this association need further exploration, these findings nonetheless underscore the need for further research into the elevated risk for cancer mortality among poultry workers. Current literature is limited on the topic, but previous studies suggest that formation of carcinogenic chemical compounds in the meat curing and smoking process can be hazardous to humans and may be worth examining in populations similar to our study that are exposed in occupational environments [33, 36]. In contrast, we observed processing poultry to be protective for esophageal cancer mortality. We hypothesize this is due to nonresponse bias in our analyses of the respondent cohort and thus, results obtained from the interview may not be valid. The impact of nonresponse bias observed in this study emphasizes the importance of including a majority of the sub‐cohort or selected controls (we obtained less than 20% of our randomly selected sub‐cohort). Future studies should establish more robust controls to ensure accurate risk estimation and minimize bias, which will improve internal validity. This study provides further evidence of a potential increased risk of esophageal cancer mortality among poultry workers. Further in‐depth studies are needed to investigate the underlying mechanisms with the goal of informing effective protection strategies for the poultry industry.

## Author Contributions

**Leanna Delhey:** concept, design, formal analysis, writing – original, review and editing. **Christina Joshua:** design, formal analysis, writing – original, review and editing. **Jaimi L. Allen:** writing – review and editing. **Robert Delongchamp:** concept, design, formal analysis. **Benjamin C. Amick:** writing – review and editing. **Wendy Nembhard:** writing – review and editing.

## Ethics Statement

The original cohort study was reviewed and approved by the Human Subjects Committee (Institutional Review Board) of the University of North Texas Health Science Center (Denton, TX, USA). The follow‐up case‐cohort study was reviewed and approved by the Institutional Review Board at the University of Arkansas for Medical Sciences (Little Rock, Arkansas, USA). Before participating, all respondents provided verbal consent agreeing to the CATI to provide information about the participant or the deceased person as appropriate.

## Conflicts of Interest

The authors declare no conflicts of interest.

## Supporting information

AJIM Supplemental Table ‐ Poultry Cohort Cancer Study 2024Nov26.xlsx.
